# Subversion of Immune Response by Human Cytomegalovirus

**DOI:** 10.3389/fimmu.2019.01155

**Published:** 2019-06-10

**Authors:** A. Raj Kumar Patro

**Affiliations:** Infectious Disease Biology Group, Institute of Life Sciences (ILS), Bhubaneswar, India

**Keywords:** HCMV (human cytomegalovirus), immune evasion, pathogenesis, superinfection, vaccine

## Abstract

Human cytomegalovirus (HCMV) is the most common cause of congenital infections and is an important pathogen in immunocompromised individuals. Despite a robust host immune system, HCMV able to replicate, evade host defenses, establish latency for life. A significant portion of HCMV genome dedicated to encode gene products for modulation of host immune response. Growing number of HCMV gene products are being recognized to play role in immune evasion. Information on viral immune evasion mechanisms by which HCMV persists in host will be useful in devising antiviral intervention strategies and development of new vaccines. This minireview provides a brief overview of immune evasion strategy adapted by HCMV by utilizing its gene products in modulation of host immune response.

## Introduction

The human cytomegalovirus (HCMV) is a ubiquitous β-herpesvirus that establishes lifelong persistent infection following introduction to an immunocompetent host. Primary infection in a healthy individual leads to mild febrile illness, whereas HCMV causes serious complications in immunosuppressed subjects, especially in transplant recipients and in immunocompromised patients (1, 2). Human cytomegalovirus is the most common cause of congenital infections leading to neurodevelopmental sequelae. Each year, 20,000–40,000 children are born with congenital human CMV infection in the US, of which 10–15% develops permanent sequelae including sensorineural hearing loss (3–5). Furthermore, substantial fraction of the asymptomatic children develops late onset hearing loss. In an attempt to reduce these disabilities and loss of life, as well as the associated economic cost, the Institute of Medicine of National Academy of Sciences, USA have ranked the development of HCMV vaccine as a highest priority (6, 7).

Decades of research on cytomegalovirus has provided novel insight in understanding the host immune response and evasion strategies adapted by the virus. HCMV has dedicated more than half of its genome encoding for modulation of host response to infection (8, 9). This mini-review article discusses on current understanding of HCMV gene products in modulation of host immune response with an emphases on the immune evasion by interference in antigen presentation and activation of NK cells, viral strain diversity and superinfection in immune subject.

## Modulation of Immune Responses by HCMV Gene Products

**Figure 1:**
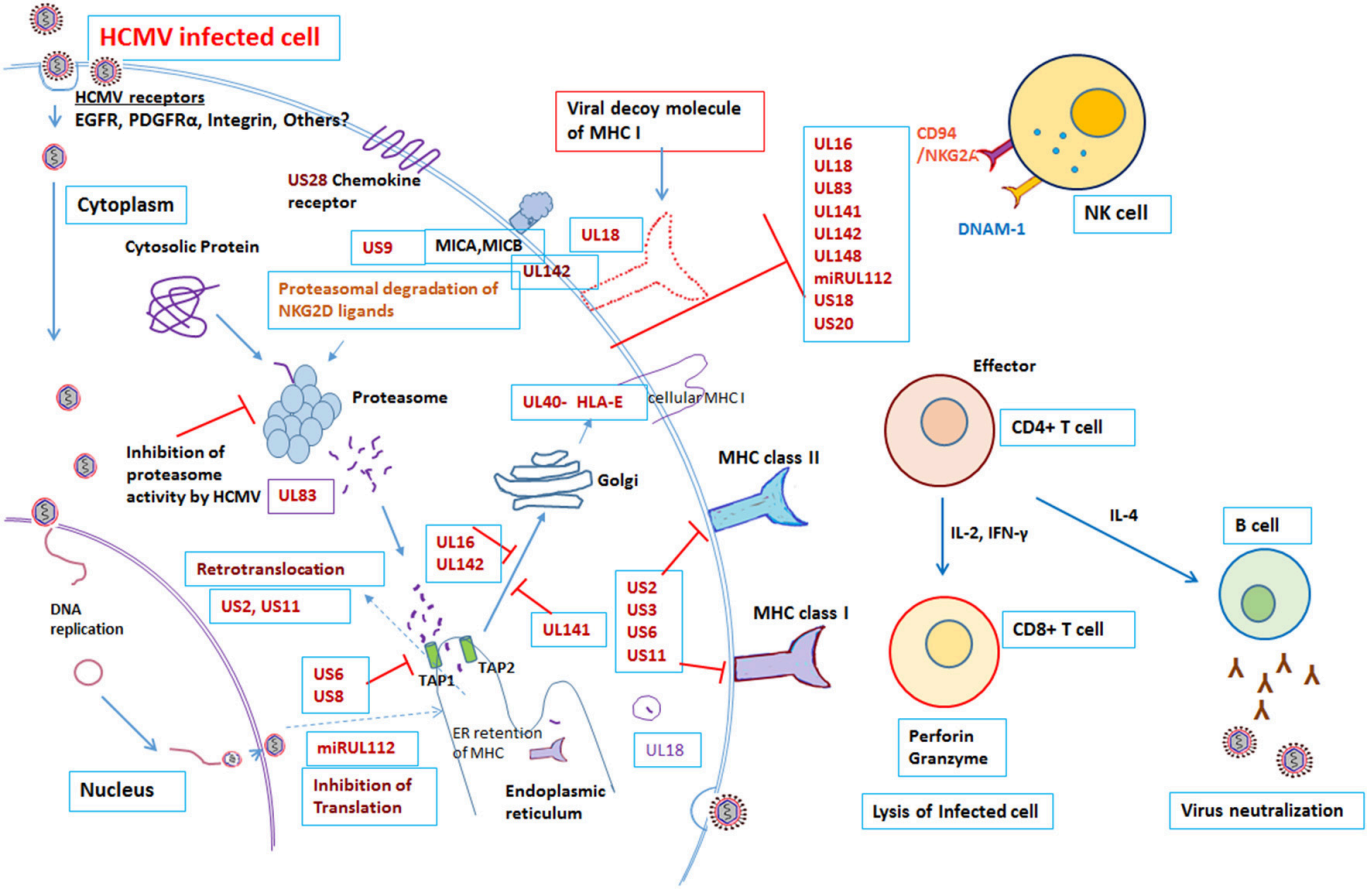
Modulation of Immune response by human cytomegalovirus. Overview of the interactions between HCMV and the immune system. Red “T” bars indicate inhibition. Blue arrows indicate activation. Detail mechanisms explained in the text.

The virus has co-evolved with its host organism for 200 million years (9, 10). HCMV has a large genome size of 236 kb with unique long (UL) and unique short (US) regions flanked by terminal repeats and internal repeats. The genome has been annotated and encodes 167 gene products, as well as non-coding RNAs, microRNAs, and with an extensive alternate mRNA splicing. However, recent report suggested that HCMV encode to have more than 750 translated ORFs (11). More than 40 HCMV gene products are recognized to have a role in modulating the host immune response following infection (12, 13). Both the innate and adaptive arms of the immune system play a crucial role in controlling HCMV infection (12, 14). Despite a robust host immune system, HCMV is able to establish latency and once infected the HCMV remains in the host for life. The virus remains latent in the myeloid progenitor cells during its dormant phase; however on stimulation, or when the immune system is suppressed, the virus can once again become active (15). The battle between the host immune system and the virus continues throughout life, with HCMV having evolved multiple mechanisms to evade the host immune response. The divergence of the immune response and incomplete viral control may be attributed to the diversity of immune modulators encoded by HCMV gene products [Figure 1, Table 1]. Many of these gene products are homologs of host genes involved in the immune response.

**Table 1:** HCMV gene products involved in modulation of host immune response.

**HCMV Gene Product**	**Effect on host immune system & mechanism of evasion**	**Reference(s)**
US2, US3, US6, US11	MHC class –I down regulation and impairment of expression; Further reduction in HCMV antigen presentation to CD8+ cells; Evasion of CD8+ T cell Immunity; Superinfection	(16, 17)
US2, HCMV Immediate Early/ Early	MHC class –II down regulation; Further reduction in HCMV antigen presentation to CD4+ cells	(12, 18)
US18 and US20	Interfere with B7-H6 surface expression involving endosomal degradation; escapes immune recognition by NK cells	(19)
UL18	Expression of human MHC class –I homolog; downregulate CTLs; Ligand decoy for NK receptors	(14, 16)
UL16	Regulation of NK cell ligand NKG2D; NK cells function impairment	(18)
UL40	NK cell evasion; HLA-E Over expression	(20)
UL83 (pp65)	IE-I sequestration; inhibit proteasome processing; Reduce action of NKp30; hinders antiviral gene expression	(21)
IE2 (immediate early) gene product	Overexpression of anti-apoptotic FLIP protein	(16, 18)
US28 (viral GPCR)	Targeting chemokine receptor; reduced inflammatory response	(12)
UL82 (pp71)	The tegument protein binds with stimulator of interferon genes to inhibit antiviral response.	(21, 22)
UL111A	HCMV encodes cmv IL-10, an homolog of human IL-10, thereby modulate immune system results in immune suppression	(23, 24)
UL141	CD155 down regulation	(14)
UL142	Inhibition of MICA	(12, 18)
UL36	Inhibition of pro-apoptotic recruitment of pro-caspase 8 to the DISC Decline in phagocytic activity (infected APCs)	(12)
UL37	Inhibition of pro-apoptotic Bcl-2 family Bak and Bax protein Apoptosis inhibition	(18)
UL97	Along with HCMV pp65 mediated immune evasion; Protein Kinase UL97 Forms a Complex with the Tegument Phosphoprotein pp65	(14, 25)
IE gene products	Induction of TGF-β: HCMV induce transcription & release of TGF-β	(26)
UL138	Latency associated; Sensitizes cells to TNF-α signaling	(15)
UL141- UL144	Encodes for homolog of TNFR; This HCMV encoded gene product inhibits cell surface expression of CD155 and CD112 (NK cell activating ligands) and the death receptor for the TNF family ligand TRAIL	(8, 14)
UL145	degradation of helicase like transcription factor- (HLTF) by recruitment of Cullin4/DDB ligase complex	(27)
UL146	Chemokine; role in inflammatory response	(14)
UL148	Suppression of CD58; Potent Modulator of CTL Function	(28)
miR-UL112	Escape from NK cell by down regulation of MICB; recognition from T cells by NKG2D decreased	(29, 30)

List of HCMV gene products involved in immune evasion.

*[US, Unique short; UL, Unique long; miR, Micro RNA; MHC, major histocompatibility complex, TAP, Transporter associated with antigen processing; NK cells, natural killer cells; CTL, cytotoxic T cell I; LIR-1, Leukocyte Immunoglobulin-like receptor 1; HLA, human leukocyte antigen; IE, Immediate early; FLIP, FLICE-inhibitory protein; FLICE, cysteine proteases (caspase-8/MACH/Mch5), CRP- C-reactive protein, MICA, MHC class I polypeptide-related sequence A; un, unknown; DISC, death-inducing signaling complex; APC, Antigen presenting cells; Bak- BCL2 Antagonist/Killer, Bax- BCL2 Associated X, Bcl-2- B-cell lymphoma 2; pp65, phospho protein 65; TGF-β, Transforming growth factor –β; TNFR, tumor necrosis factor receptor; Cullin4/DDB, Cullin-4A·DNA Damage-binding Protein; CD, cluster of differentiation]*.

To eliminate the virus, the host needs to have an effective immune system. After viral infection, host antigen presenting cells must present viral antigen to the immune cells in order to stimulate effector cells to eliminate the virus. However, HCMV has devised strategies to limit this presentation. NK cells are normally responsible for immediate control of viral infections; however, there are number of HCMV gene products that block NK cell mediated recognition. Approximately, there are 12 HCMV gene products, US20, UL16, UL17, UL18, UL40, UL43, UL140, UL83, UL141-UL144, and UL148, known to control NK cell modulation (Table 1). HCMV UL16, UL17, UL40, UL140, and UL142 genes all encode products that down-regulate NK cell activity by imitating the host HLA class I. For example, UL40 encodes a canonical ligand for HLA-E and negatively regulates NK cells, which results in down-regulation of activating ligand CD155 (20). Individuals with impaired NK cell function, succumbs to severe herpesvirus infections (31). In addition, HCMV gene products UL18 and UL83 (pp65) encode for an MHC-I homolog, modulate expression of other HCMV genes and inhibit NK cell lysis (12, 20). Furthermore, the HCMV microRNA miR-UL122 acts to suppress host MICB surface expression (13, 20, 29).

As is a common characteristic of herpes viruses, HCMV is able to interfere with the class I MHC molecule involved in antigen presentation to CD8+ T cells. HCMV establishes persistent infection by producing host homologous molecules that prevent recognition and interfere with antigen presentation, subverting the cytotoxic T lymphocytes (CTLs). Viral antigens are normally presented by the MHC class I proteins on the infected cell surface. HCMV gene products obstruct peptide translocation to the ER lumen and stimulate degradation of the MHC class I proteins before they can reach the cell surface. For example, the HCMV US3 gene product degrades the MHC class I heavy chain by interacting with Tapasin and retaining the class I molecule at the site of synthesis, in the ER. In addition, the US2 and US11 gene products relocate the heavy chain of MHC class I into the ER for proteosomal degradation. Similarly, another gene product of HCMV, US6, prevents peptide loading by inhibiting the binding of ATP to TAP, thereby preventing the transport of peptides through the TAP pore. The combined functions of the HCMV gene products US2, US3, US6, and US11, therefore, lead to peptide transport blockade, retention of MHC class I in the ER and ultimately proteasomal degradation. In addition, the gene product US2 interferes with MHC class II signal transduction by degradation of MHC class II proteins. US2 targets the class II DR and DMα chains for degradation in the cytosol, thereby preventing antigen presentation to CD4+ T lymphocytes (12, 14, 16, 32).

In addition to the above, the HCMV UL83 gene product, pp65 blocks the processing of immediate early-1 in the proteasome by phosphorylation. Besides, the tegument Protein UL82 evades antiviral immunity by inhibiting stimulator of interferon (STING) signaling (21) and may be responsible for induction of latency (15, 22). Recently, Nightingale et al. reported that the HCMV gene product UL145 facilitates degradation of the antiviral factor helicase like transcription factor (HLTF) by recruiting the host Cullin4 E3 ligase complex, and captures Cullin3 to invoke the strategy of immune evasion (27). Additionally, the HCMV late gene product UL111A encodes cmvIL-10, a homolog of human IL-10, which is expressed during viral latency, and causes a state of immune suppression (23). The cytokine Interleukin-10 has an immunosuppressive role on several effector cells of the immune system. The HCMV gene product cmvIL-10 exerts an immunosuppressive effect on the host by modulating the expression of the MHC class I and II molecules and interfering with dendritic cell (DC) function (24). In a murine model of CMV, following productive infection with CMV both *in vitro* and *ex vivo*, the virus reduced the expression of MHC as well as co-stimulation of DC. This eventually led to loss of expression of IL-2 and IL-12 and hindrance of DC differentiation (33–35). A recent report by Wang et al also demonstrated that the HCMV UL148 gene product suppresses co-stimulation and expression of the cell adhesion molecule CD58, endorsing cellular immune defense evasion by impairing NK and T cell activation (28). This work was further supported by HCMV UL148 mediated tropism and immune evasion by unfolded protein response (36). In Rhesus model, Rh159, a homolog of HCMV UL148 involved in retention of distinct set of costimulatory molecules and involved in NK cell evasion (37). HCMV UL148 gene products encode for avoidance of killing of HCMV infected cells from NK cells by down regulating MICA (38).

HCMV possesses a unique challenge, as it is able to super-infect in a subject already infected with the virus, even in the presence of a strong specific immune response. Several studies have demonstrated congenital HCMV infection in offspring of immune mothers because of reinfection with a different strain of virus (39–43). Further, congenital infected infant born to immune mother may develop sequelae similar to infants born to mother with primary infection during pregnancy. It has also been observed that infection with more than one strain of HCMV is common in nature (39, 40, 44). HCMV strain polymorphism could contribute to immune evasion. Since HCMV glycoproteins are highly polymorphic, antibody response to one strain may not efficiently neutralize infection with a different strain and this could enable to superinfection (45–47). In addition to interference in antigen presentation, the CMV gene products US2, US3, US6, and US11 encode for human homologs that interfere with the function of CD8+ T cells. This allowed viral replication and super-infection with a different strain of virus in a rhesus macaque model. This was confirmed, by the observation that US2-11 mutant virus, although able to produce infection, was unable to super-infect (17). However, further studies are needed to decipher the detailed mechanisms of the CTL response in contending with the combined action of these HCMV gene products. The large genome size of HCMV enables it to utilize an array of genes for host immune evasion, which allows long-term association and adaption of the virus in the host. In an immunocompetent host, viral latency is critical for its survival. After primary infection, the virus persists for a lifetime regardless of pre-existing immunity. During latency, the viral genome is maintained in the host without active replication and retains the capacity to reactivate in response to activation signals (48). Studies have linked various latency-associated determinants to HCMV latency (15), however, the detailed mechanisms of immune evasion during latency and how the virus persists in the host for life remains elusive. Deciphering these mechanisms could provide clues to allow us to prevent reactivation of this latent virus in congenital and transplant setup. Further, a note of caution is required; HCMV is strictly species specific. Since much of our understanding on cytomegalovirus biology is derived from *in vitro* cell culture studies and animal models, it is necessary to test these immune evasion functions in the appropriate setting. For instance, the UL18 gene product of HCMV encoding an MHC class I homolog was proposed to block NK cell activity by binding with KIR receptors; however, later studies have found it to enhance killing of infected fibroblasts by NK cells (12).

Further, extensive genetic variability has been observed in clinical isolate of HCMV (4, 40, 49–51), and even within a single host (4, 44, 52–54). High throughput sequencing of HCMV clinical isolates reveals that intrahost HCMV populations were as variable as seen in RNA virus quasispecies (52, 53). Viral strain diversity, differences in culture systems and population heterogeneity, make the generalization of genetic information difficult. In addition, a recent report showed that HCMV seroprevalence is related to a shift in immune phenotype along an age axis (55). This immunotypes varies in younger vs. elder individuals (55–57). In due course of evolution with the host, HCMV has been significant in shaping host immune system (57). HCMV also affects the host in response to infection with other pathogen. In HCMV seropositive children and in aging individuals have negative impact to Influenza; however, in younger individuals HCMV infection enhance immune response to influenza (58). Viral strain diversity could limit effective antiviral function, and the evasion strategy adapted by HCMV further complicates the development of an effective vaccine (45, 59). This underscores the need for large-scale genetic and immunological profiling studies, which could provide a decisive correlation on the nature of protective immune responses (56, 59–61).

HCMV has devised multiple strategies to interfere with antigen presentations and escape from CTL response, but this does not abrogate with the development of CTL response by host. This underscores the critical role of CD8+ T cells in HCMV infected cells as targets for immune clearance. Studies from adaptive transfer of HCMV specific CTL, in bone marrow transplant subjects, provide protection from HCMV disease (62). The complex interaction between the HCMV immune-evasins and host factors contributes to the levels of viral persistence in host (63). Information on viral immune evasion mechanisms by which HCMV persists in host will be useful in devising antiviral intervention strategies and development of new vaccines. Deletion of immune evasions could be a novel strategy for virus attenuation for vaccine candidate without compromising CD8 T cell response (64). Hansen et al reported that Simian immunodeficiency virus (SIV) protein expressing rhesus cytomegalovirus vector elicits SIV specific CD8+ T cells which recognizes unusual, diverse epitopes and results in immune clearance (65, 66). Thus, CMV vectors, genetically altered for diverse CD8+ T cell response could be useful for effective prophylactic and therapeutic vaccination (9, 65–68). Further, this could be useful in ultimately designing an effective vaccine that could protect primary as well as reinfections.

## Conclusions

In conclusion, human cytomegalovirus is a master of disguise. HCMV has evolved mechanisms to replicate and evade the host immune system by targeting the host cell machinery. Information on the host cell receptor targeted by this virus and the mechanisms utilized to operate cellular processes and evade the host immune system will provide clues to viral pathogenesis. An increasing number of HCMV gene products have been reported to play roles in immune evasion. These gene products sophistically orchestrate to modulate the host immune system, thereby allowing persistent and latent infection and life-long existence in the host. Information on viral escape mechanisms will be useful in rational design of antiviral drugs and should bring us one step closer to development of an effective vaccine.

## Author Contributions

The author confirms being the sole contributor of this work and has approved it for publication.

### Conflict of Interest Statement

The author declares that the research was conducted in the absence of any commercial or financial relationships that could be construed as a potential conflict of interest.
